# Harnessing Brazilian biodiversity database: identification of flavonoids as potential inhibitors of SARS-CoV-2 main protease using computational approaches and all-atom molecular dynamics simulation

**DOI:** 10.3389/fchem.2024.1336001

**Published:** 2024-02-22

**Authors:** João Augusto Pereira da Rocha, Renato Araújo da Costa, Andreia do Socorro Silva da Costa, Elaine Cristina Medeiros da Rocha, Anderson José Bahia Gomes, Alencar Kolinski Machado, Solange Binotto Fagan, Davi do Socorro Barros Brasil, Anderson Henrique Lima e Lima

**Affiliations:** ^1^ Laboratory of Modeling and Computational Chemistry, Federal Institute of Education, Science and Technology of Paraná (IFPA) Campus Bragança, Bragança, Brazil; ^2^ Laboratório de Planejamento e Desenvolvimento de Fármacos, Instituto de Ciências Exatas e Naturais, Universidade Federal do Pará, Belém, Brazil; ^3^ Laboratory of Biosolutions and Bioplastics of the Amazon, Graduate Program in Science and Environment, Institute of Exact and Natural Sciences, Federal University of Pará (UFPA), Belém, Brazil; ^4^ Graduate Program in Chemistry, Institute of Exact and Natural Sciences, Federal University of Pará, Belém, Brazil; ^5^ Laboratory of Molecular Biology, Evolution and Microbiology, Federal Institute of Education Science and Technology of Paraná (IFPA) Campus Abaetetuba, Abaetetuba, Brazil; ^6^ Graduate Program in Nanosciences, Franciscana University, Santa Maria, Brazil

**Keywords:** SARS-CoV-2, main protease, flavonoids, drug-likeness, natural products, molecular docking, molecular dynamics, MMPBSA

## Abstract

SARS-CoV-2 (Severe Acute Respiratory Syndrome Coronavirus 2) is the etiological agent responsible for the global outbreak of COVID-19 (Coronavirus Disease 2019). The main protease of SARS-CoV-2, Mpro, is a key enzyme that plays a vital role in mediating viral replication and transcription. In this study, a comprehensive computational approach was employed to investigate the binding affinity, selectivity, and stability of natural product candidates as potential new antivirals acting on the viral polyprotein processing mediated by SARS-CoV-2 Mpro. A library of 288 flavonoids extracted from Brazilian biodiversity was screened to select potential Mpro inhibitors. An initial filter based on Lipinski’s rule of five was applied, and 204 compounds that did not violate any of the Lipinski rules were selected. The compounds were then docked into the active site of Mpro using the GOLD program, and the poses were subsequently re-scored using MM-GBSA (Molecular Mechanics Generalized Born Surface Area) binding free energy calculations performed by AmberTools23. The top five flavonoids with the best MM-GBSA binding free energy values were selected for analysis of their interactions with the active site residues of the protein. Next, we conducted a toxicity and drug-likeness analysis, and non-toxic compounds were subjected to molecular dynamics simulation and free energy calculation using the MM-PBSA (Molecular Mechanics Poisson-Boltzmann Surface Area) method. It was observed that the five selected flavonoids had lower MM-GBSA binding free energy with Mpro than the co-crystal ligand. Furthermore, these compounds also formed hydrogen bonds with two important residues, Cys145 and Glu166, in the active site of Mpro. Two compounds that passed the drug-likeness filter showed stable conformations during the molecular dynamics simulations. Among these, NuBBE_867 exhibited the best MM-PBSA binding free energy value compared to the crystallographic inhibitor. Therefore, this study suggests that NuBBE_867 could be a potential inhibitor against the main protease of SARS-CoV-2 and may be further examined to confirm our results.

## Introduction

In late 2019, a previously unknown zoonotic disease called Coronavirus Disease 2019 (COVID-19) surfaced as a viral respiratory infection in Wuhan, China. Coronavirus 2 (SARS-CoV-2), rapidly spreading worldwide. In March 2020, the World Health Organization (WHO) declared this infectious disease a pandemic (Chauhan, 2020). As of 25 October 2023, there have been 771,549,718 confirmed cases of COVID-19, resulting in 6,974,473 deaths worldwide, according to the WHO (https://covid19.who.int, accessed on 27 October 2023). Although COVID-19 vaccines have been developed remarkably quickly, it remains uncertain how well they will perform in the long term and whether they will be effective against potential future variants of SARS-CoV-2 and other coronaviruses (Heaton, 2020; Jackson et al., 2020; Yu et al., 2020; Panda et al., 2023). With commercially available antiviral treatments proving to be extremely ineffective (Liu et al., 2022; Purohit et al., 2023), the need for the search for potentially more effective antivirals arises.

The primary goal of antiviral drug research for SARS-CoV-2 is to target the viral entry mechanism or viral genome replication (Asselah et al., 2021). SARS-CoV-2 possesses an RNA genome encoding two polyproteins, pp1a and pp1ab, four structural proteins, and various accessory proteins (Qiao et al., 2021). The main protease of SARS-CoV-2 (Mpro, also known as 3CL protease) cleaves the polyproteins at 11 different sites to release mature non-structural proteins (nsps), which play crucial roles in viral RNA replication and immune evasion (Jin et al., 2020; Owen et al., 2021; Wu et al., 2022). Inhibiting Mpro would essentially disrupt viral proliferation throughout the human body (Zhang et al., 2020; Singh et al., 2022). Considering the essential role of Mpro in maturing nsps and SARS-CoV-2 replication, combined with the absence of known human proteases with similar cleavage specificity, makes it an attractive target for the development of antivirals without adverse effects (Qiao et al., 2021; Hu et al., 2022; Liu et al., 2022; Huang et al., 2023). Several SARS-CoV-2 Mpro inhibitors have undergone clinical trials, and nirmatrelvir and ensitrelvir have received regulatory approval (Owen et al., 2021; Mukae et al., 2022; Mukae et al., 2023). However, mutations conferring resistance to nirmatrelvir and ensitrelvir have already been identified in SARS-CoV-2 Mpro (Heilmann et al., 2022), highlighting the importance of seeking new Mpro inhibitors.

Due to the urgent need to find an effective therapy to combat SARS-CoV-2, several studies have emphasized the importance of Natural Products (NPs) as potential antiviral agents (Antonio et al., 2020; Islam et al., 2020; Mani et al., 2020; Wang and Yang, 2020; Rubio-Martínez et al., 2021). Furthermore, some research has focused on the use of NPs as inhibitors of the main protease of SARS-CoV-2 (Mpro) (Aanouz et al., 2021; Ray et al., 2022; Ahamed and Arif, 2023; Purohit et al., 2023).

Among readily accessible Natural Products (NPs), flavonoids stand out, which are naturally occurring polyphenolic compounds found in fruits and vegetables, and have demonstrated significant antiviral activities (Zakaryan et al., 2017; Cataneo et al., 2019; Wang and Yang, 2020). These compounds play a relevant role in enhancing the host’s defense system against viral infections, reducing infection, and inhibiting cytokine generation (Jo et al., 2020; Solnier and Fladerer, 2021; Alzaabi et al., 2022). The potential of flavonoids as antiviral agents against SARS-CoV-2 is reported in the literature (Cherrak et al., 2020; Wang and Yang, 2020; Gogoi et al., 2021; Liskova et al., 2021; Owis et al., 2021; Alzaabi et al., 2022; Liu et al., 2022; Chatterjee et al., 2023). Jo and co-workers (Jo et al., 2020) identified a series of flavonoids as effective inhibitors of SARS-CoV-2 replication in in vitro experiments. Furthermore, Mohapatra and colleagues discussed the specific importance of the Cys145 residue in likely covalent interactions with natural products (Mohapatra et al., 2023). Recently, in a SARS-CoV-2 replication assay and molecular dynamics studies, flavonoids were identified as inhibitors of Mpro (Lin et al., 2023). Recent reviews highlight flavonoids with confirmed *in vitro* anti-SARS-CoV-2 Mpro activity (Solnier and Fladerer, 2021; Antonopoulou et al., 2022). Therefore, exploring flavonoids as potential inhibitors of SARS-CoV-2 Mpro emerges as a promising strategy in the search for alternative therapies based on natural products.

The present study aimed to employ computational approaches to screen flavonoids derived from Brazilian biodiversity with the purpose of identifying potential inhibitors of the main protease (Mpro) of SARS-CoV-2. The results of this study will guide future efforts in identifying and developing Mpro inhibitors based on natural flavonoids. Additional experimental studies will be necessary to validate computational predictions and assess the activity of these compounds in cellular models.

## Materials and methods

### Flavonoid-based virtual screening for SARS-CoV-2 protease inhibitors

Initially, a library containing 288 flavonoids obtained from the Natural Products database extracted from Brazilian biodiversity (NuBBEDB) was used in the virtual screening (Valli et al., 2013; Pilon et al., 2017). Subsequently, all compound structures were filtered based on the Lipinski “Rule of 5” (RO5) physicochemical filter (Lipinski et al., 2001) to obtain drug-like molecules. Compounds that did not violate Lipinski’s rule were considered for the virtual screening study.

The 3D crystal structure of SARS-CoV-2 Mpro with PDB ID 6M2N complexed with the crystallographic inhibitor Baicalein (3WL) was used for virtual screening (Su et al., 2020). The Chimera software was used for protein preparation, removing the crystallographic ligand 3WL and water molecules. Hydrogen atoms were added, and protonation states at pH 7.0 were assigned to the protein using the H++ server (Gordon et al., 2005).

The GOLD program (Verdonk et al., 2003) was used to perform molecular docking simulations. First, the crystallographic ligand 3WL was re-docked, and the RMSD between the crystallographic ligand and the generated pose was calculated to validate the molecular docking protocol. The docking spatial search docking sphere was defined using the coordinates of the 3WL ligand in the protein’s crystallographic structure with a radius of 10 Å using the GoldScore scoring function (Verdonk et al., 2003). After the validation step, natural products from the flavonoid classes were subjected to molecular docking following the same protocol as validation. Subsequently, the poses were rescored according to the workflow proposed by Harutyun Sahakyan, 2021; Sahakyan, 2021), which uses the iPBSA code to reclassify the poses generated in molecular docking through the calculation of binding free energy using the MM-PB (GB) SA methods.

For the calculation of MM-GBSA binding free energy, protein and ligand parameterization was performed first, then the complexes were minimized, and finally, MM-GBSA calculations were performed using AmberTools23 (Case et al., 2023). Ligand charges were calculated using the AM1-BCC method ((Jakalian et al., 2002). The ff14SB force field was used to describe protein parameters, and the General Amber Force Field (GAFF) for ligands (Wang et al., 2004; Maier et al., 2015). Required input files (coordinates and topologies) with mbondi3 radii were prepared using tLEaP. Minimization was carried out in generalized implicit solvent models for each protein-ligand complex using the sander mechanism (Salomon-Ferrer et al., 2013). For each complex, the maximum number of minimization cycles was set at 1,000, with 500 steps using steepest descent and another 500 steps using the conjugate gradient algorithm. The final snapshot of the minimized protein-ligand complex was used to rescore the poses through MM-GBSA binding free energy calculation.

### Molecular dynamics simulation

The compounds that passed the toxicity analysis were then subjected to a molecular dynamics (MD) simulation study to analyze the stability and potential conformational changes of the target protein complexed with flavonoids using Amber22 (Costa et al., 2023).

The structures of the protein and ligands were treated using the Amber ff14SB and the general Amber force field (GAFF), respectively. For MD, the systems were solvated in the tLeap module in a truncated octahedral box with a 12Å edge and filled with TIP3P water (Price and Brooks, 2004). Counter ions were added as needed to neutralize the systems. Subsequently, the systems were minimized in four steps, gradually removing position restraints on atom groups. The minimization process starts with the steepest descent algorithm and switches to the conjugate gradient. Next, slow heating for 500 ps to 300 K at constant volume with position restraints on the solute and unrestricted equilibration for 500 ps at constant pressure were performed. Using a collision frequency of 2 cm^−1^ and linked to a Langevin thermostat, the temperature was maintained at 300 K. The SHAKE algorithm (Ryckaert et al., 1977) and Particle Mesh Ewald (PME) (Darden et al., 1993) were used to limit bond lengths involving hydrogen atoms, and a 10 Å cutoff was applied for non-bonded interactions. Finally, a 100 ns production run was conducted without positional restraints at a constant temperature of 300 K.

### Binding free energy calculations

To describe the binding affinity of the natural products and the crystallographic inhibitor with the Mpro protein, we performed binding free energy calculations using the MM/PBSA method (Genheden and Ryde, 2015) available in the AmberTools23 package (Case et al., 2023), while the mathematical description was published in previous studies (Costa et al., 2022). The last 10 ns of the MD simulation trajectories were used to calculate the binding free energy and the decomposition of the binding free energy.

### Toxicity analysis

The five flavonoids were analyzed for different types of toxicities, including tumorigenic, mutagenic, reproductive effectiveness, irritant, and drug-likeness using ORISIS Data Warrior 5.2.1 (Sander et al., 2015).

## Results and discussion

### Virtual screening and molecular docking

Although some natural products (NPs) have bioactivity or drug-like activity, many NPs have low solubility or chemical instability, making their transformation into parenteral drugs challenging. Additionally, the complex structures of many NPs result in high molecular weights, which are likely to have negative effects on intestinal absorption (Li et al., 2019).

In this study, the bioavailability of the 288 flavonoids was evaluated based on their physicochemical properties using the Lipinski’s rule. This step was carried out within the NuBBEDB. It was found that 204 flavonoids did not violate any of the Lipinski’s rules and were therefore selected for the virtual screening phase.

**FIGURE 1:**
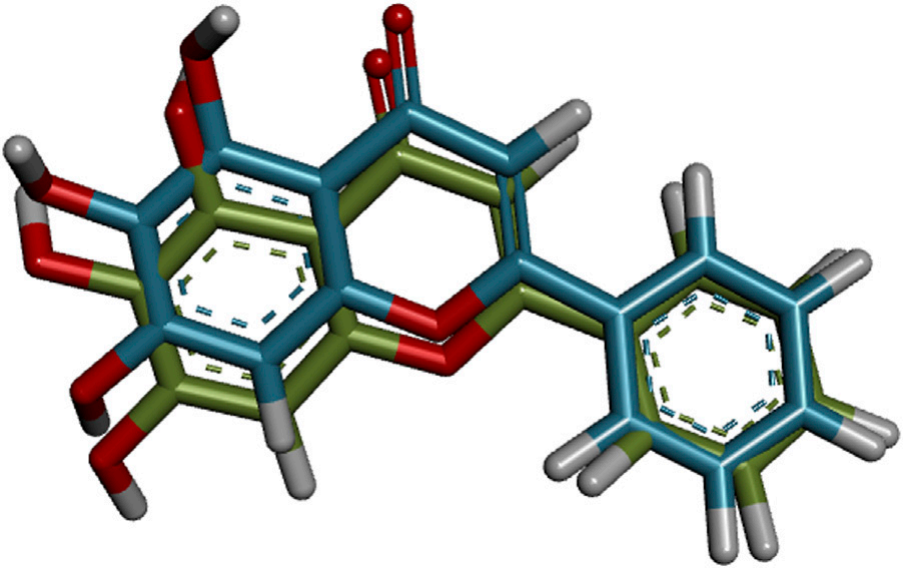
Validation of molecular docking protocols using the GOLD program for the crystal structure of the COVID-19 main protease (Mpro) in complex with 3WL inhibitor. Blue is the co-crystal ligand and green is the docking pose.

Before the virtual screening of the flavonoids, the molecular docking protocol was validated by redocking the co-crystallized ligand 3WL into the active site of Mpro. It was found that the redocked conformation of the ligand perfectly overlapped with the co-crystallized ligand, with an RMSD value of 0.636 Å (Supplementary Figure S2). This result suggests that the GOLD program exhibits satisfactory accuracy in repositioning the 3WL ligand within the active site of Mpro. The docking protocol is considered satisfactory when the RMSD value between the docking pose and the crystallographic ligand pose is less than 2.0 Å (Wang et al., 2016; Costa et al., 2023). Figure 1 illustrates the overlapping alignment of the redocked pose and the crystallographic ligand.

The validated docking protocol was subsequently used for virtual screening based on molecular docking of 204 flavonoids extracted from the Brazilian biodiversity available in NuBBEDB (Pilon et al., 2017). After being scored by the GoldScore scoring function in the GOLD program, the poses were rescored using the method of binding free energy (∆Gbind) calculation MM/GBSA using AmberTools23.

Virtual screening has become a widely applied method in early-stage enzymatic inhibitor discovery projects. The success in predicting hit candidates is closely related to the scoring function applied. However, docking scores have limitations as they often show a weak correlation with experimental results, with only a small fraction of hits exhibiting activity in in vitro validation assays (Rastelli and Pinzi, 2019). Because of this, in our study, we applied the rescoring method using MM/GBSA binding free energy calculations as it is a more accurate method for the selection of the best molecules in virtual screening studies (Rastelli and Pinzi, 2019; Poli et al., 2020; Sahakyan, 2021).

After molecular docking and rescoring of the poses, the top five ranked flavonoids based on the MM/GBSA values were selected, and the interactions of these structures with the amino acid residues in the active site of the Mpro protein were analyzed and compared with those of the crystallographic ligand 3WL, used as a reference compound in this study.

Table 1 shows the ∆Gbind MM/GBSA values of the top five rescored compounds. A- Nubbe_867 (de Sousa et al., 2014); B- Nubbe_1884 (Braz Filho and Gottlieb, 1971); C- Nubbe_1310 (dos Santos et al., 2009); D- Nubbe_1890 (Braz Filho and Gottlieb, 1971); E- Nubbe_2328 (Ferracin et al., 1998), as well as the reference inhibitor 3WL. Supplementary Table S1 shows the ∆Gbind values obtained by the MM/GBSA method for all flavonoids used in this study.

**TABLE 1:** Binding free energies of the top 5 rescored hits along with the co-crystallized ligand 3WL obtained using the MM/GBSA method.

The compounds with best scores	Binding free energy (kcal mol^-1)^
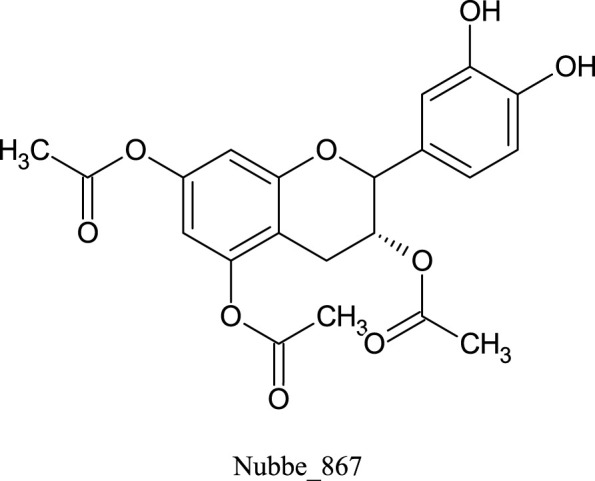 Nubbe_867	−51. 57
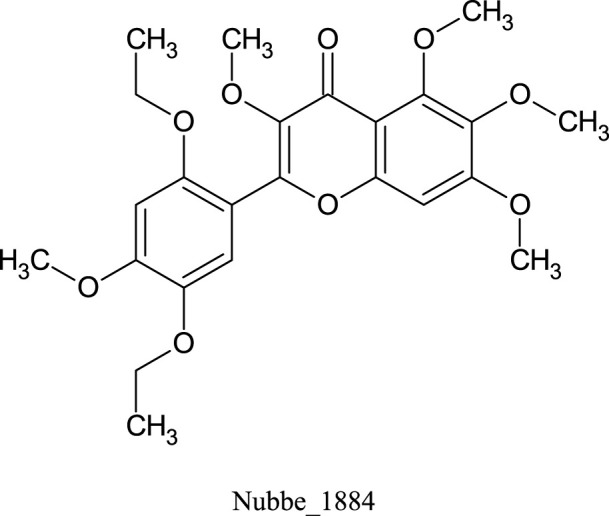 Nubbe_1884	−50. 12
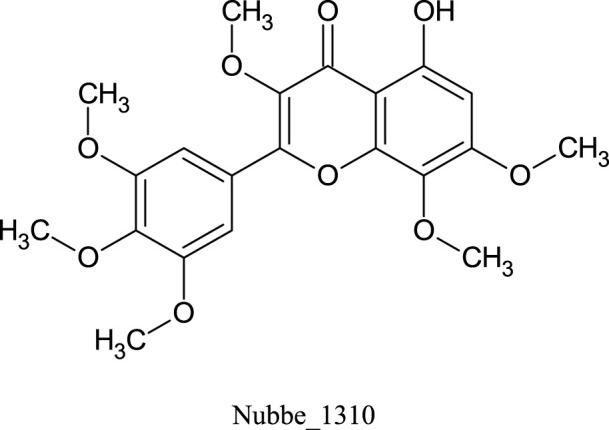 Nubbe_1310	−49. 55
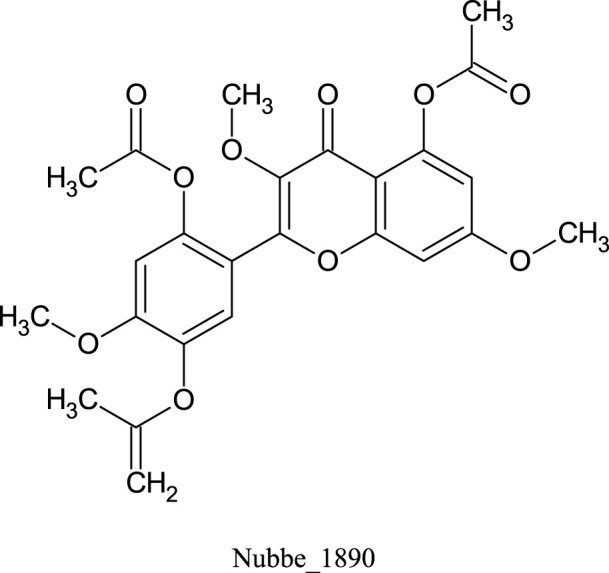 Nubbe_1890	−49. 55
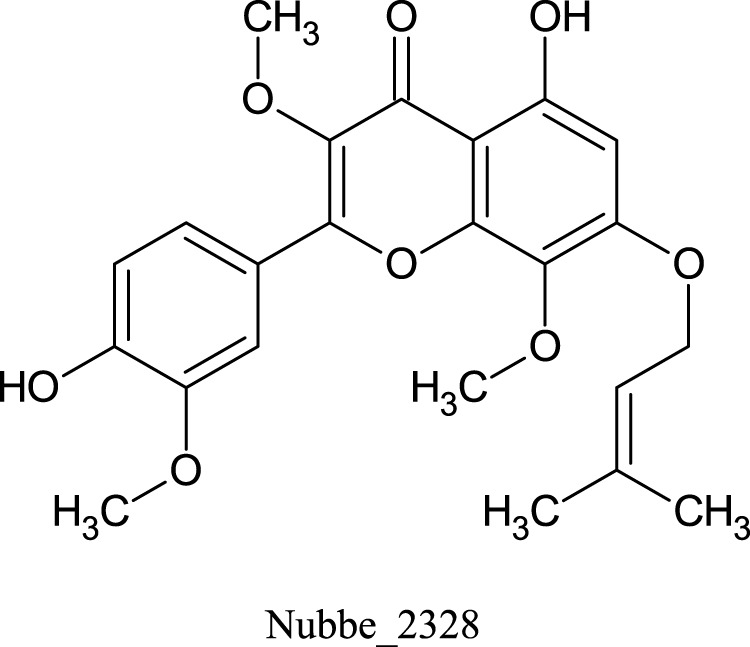 Nubbe_2328	−47. 08
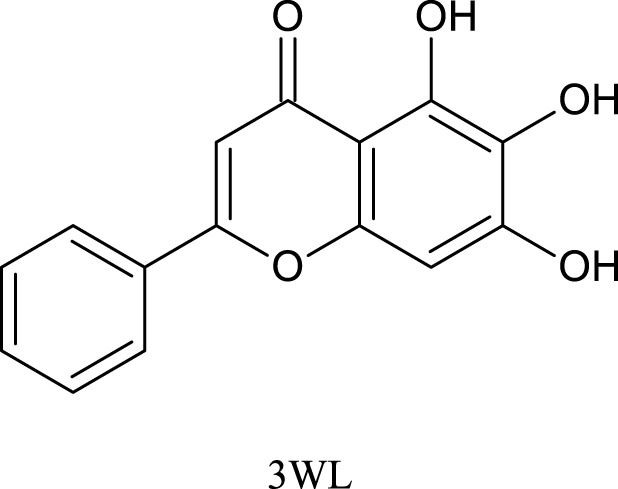 3WL	−31. 26

The analysis of the new MMGBSA scores, used to avoid false predictions, revealed that the five selected compounds have better binding affinities with the Mpro enzyme, ranging from −51.57 to −47.08 kcal mol^−1^, when compared to the reference inhibitor (3WL: −31.26 kcal mol^−1^), as shown in Table 1.

**FIGURE 2:**
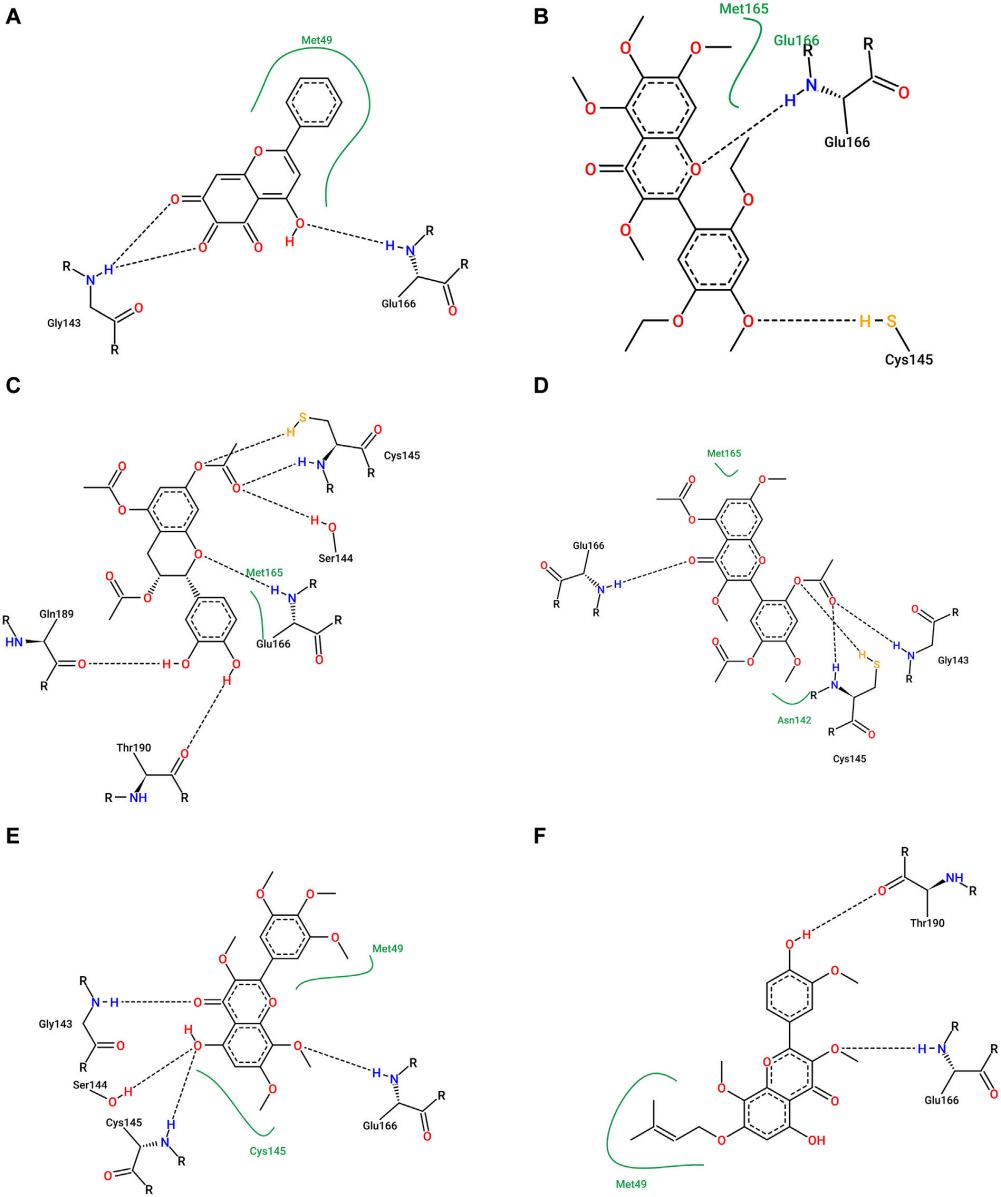
Figure 1: **(A)** 3WL, **(B)** NuBBE_1884, **(C)** NuBBE_867, **(D)** NuBBE_1890, **(E)** NuBBE_1310, and **(F)** NuBBE_2328 binding interactions with Mpro. Dashed lines indicate hydrogen bonds between the ligands and interacting Mpro residues.

We analyzed the binding modes of these compounds to the active site of the Mpro enzyme. The interactions made by the compounds and the inhibitor 3WL are shown in Figure 2. Our results demonstrated that the selected compounds formed an equal or greater number of hydrogen bonds with the target protein compared to the crystallographic inhibitor 3WL. This explains the higher binding affinity of these flavonoids to the Mpro protein than 3WL (Gogoi et al., 2021).

The analysis of the residues involved in the interactions between the compounds and Mpro showed that NuBBE_867 interacted with the residues Ser144, Cys145, Glu166, Gln192, Gln149, and Thr190. NuBBE_1310 formed interactions with the residues Cys145 and Glu166. NuBBE_1884 interacted with Gly143, Ser144, Cys145, and Glu166. NuBBE_1890 interacted with the residues Gly143, Cys145, and Glu166, and NuBBE_2328 interacted with Glu166 and Thr190. All of these residues are part of the protein’s active site and are responsible for stabilizing these ligands (Su et al., 2020).

The flavonoids NuBBE_867, NuBBE_1310, NuBBE_1884, and NuBBE_1890 form hydrogen bonds with the important catalytic residue Cys145, which is part of the S1 binding pocket present in the catalytic domains I and II of the SARS-CoV-2 Mpro. Along with residue His41, they form the catalytic dyad within the substrate-binding site that actively participates in the catalytic function of the protein. Therefore, binding of these flavonoids to this residue may reduce the catalytic activities of Mpro, ultimately leading to a reduction in viral replication (Gogoi et al., 2021).

Another important residue that interacts with all selected flavonoids is Glu166, a key amino acid involved in the dimerization of the Mpro protein and assists in creating the S1 substrate binding pocket (Goyal and Goyal, 2020; Silvestrini et al., 2021). The formation of a hydrogen bond with the Glu166 residue is reported in the study by Duong and Nguyen (Duong and Nguyen, 2023) as one of the key interactions of non-covalent Mpro inhibitors. It is interesting to note that the crystallographic inhibitor 3WL, used as a reference in this study, also formed a hydrogen bond with the Glu166 residue, which corroborates our results (Su et al., 2020).

The residues Cys145 and Glu166 have been recently reported in molecular docking studies of inhibitors involving natural flavonoids as key residues in the binding process at the Mpro site (Cherrak et al., 2020; Su et al., 2020; Lin et al., 2023), and also with different compounds, corroborating with our results (Gahlawat et al., 2020; Kumar et al., 2020; Gogoi et al., 2021; Ngo et al., 2021; Patel et al., 2021; Antonopoulou et al., 2022; Salarizadeh et al., 2022; Ahamed and Arif, 2023; Rampogu et al., 2023). It is important to mention that all ligands fit perfectly into the protein’s binding pocket, revealing that all ligands form a stable complex with the target receptor.

### Toxicity analysis

The five flavonoids were subjected to drug-likeness evaluation and assessment of mutagenic, tumorigenic, reproductive effective, and irritant parameters using ORISIS Data Warrior. It was observed that two flavonoids, NuBBE_867 and NuBBE_1890, showed no toxicity based on the toxicity parameters used in the study. On the other hand, NuBBE_1310 exhibited mutagenic and tumorigenic properties, while NuBBE_2328 displayed mutagenic, tumorigenic, and irritant properties. NuBBE_1884 showed a negative drug-likeness probability. The results of toxicity prediction and drug-likeness property analysis are presented in Table 2. Based on the data obtained from ORISIS Data Warrior (Sander et al., 2015), compounds with higher or positive drug-likeness probability values are considered good drug candidates. Since compounds NuBBE_1310 and NuBBE_2328 exhibited toxic effects, and NuBBE_1884 had a negative drug-likeness probability, they were not considered for further analysis. The non-toxic compounds, NuBBE_867 and NuBBE_1890, underwent molecular dynamics simulation studies.

**TABLE 2:** Toxicity and drug-likeness analysis.

Compound	Drug likeness	Mutagenic	Tumorigenic	Reproductive efective	Irritan
NuBBE_867	02,585	none	none	none	none
NuBBE_1310	−0,1051	high	high	none	none
NuBBE_1884	−0,9442	none	none	none	none
NuBBE_1890	06,193	none	none	none	none
NuBBE_2328	00,517	high	high	none	low
3WL	028,194	none	none	none	none

### Molecular dynamics simulation

Molecular Dynamics (MD) simulation was performed to assess the flexibility, and stability of the protein-ligand complex, and the interactions of ligands with the SARS-CoV-2 Mpro protein. In this study, MD simulations were conducted for the Mpro-NuBBE_867 and Mpro-NuBBE_1890 complexes, selected based on toxicity and drug-likeness analyses, as well as the Mpro-3WL (reference inhibitor) complex and the unbound form of the protein (Apo). To predict the dynamic behavior and structural changes in the complexes, MD simulations were carried out over a 100 ns and the resulting trajectories were used to investigate complex RMSD, RMSF, and the number of hydrogen bonds.

**FIGURE 3:**
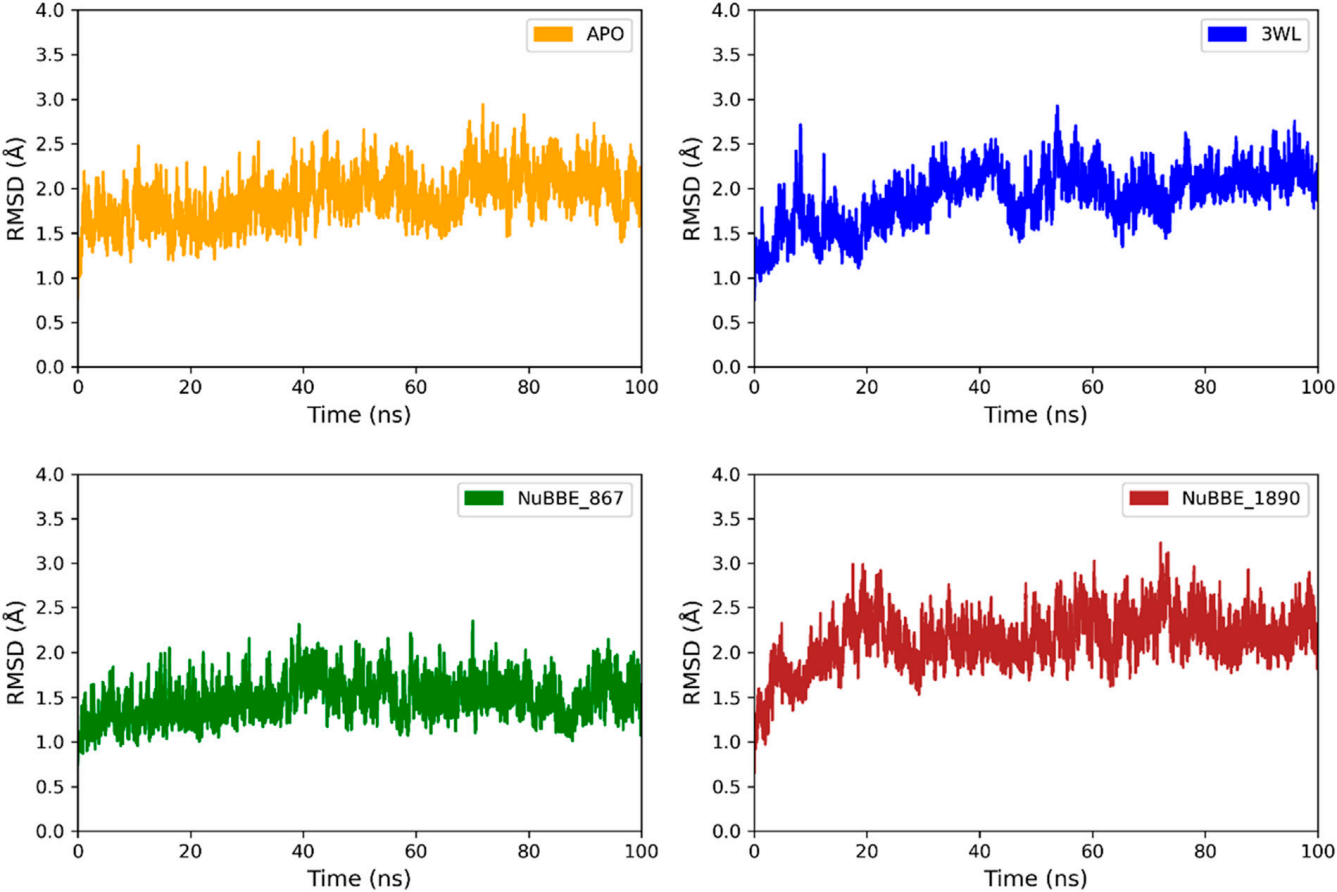
RMSD of the Apo form of Mpro and the Mpro complexes with 3WL, NuBBE_867, and NuBBE_1890 over a 100 ns MD simulation time.

RMSD analysis was performed to monitor the conformational stability of the ligand-protein complexes (Mpro). The RMSD plots for the ligand-protein complexes (Mpro-NuBBE_867, Mpro_NuBBE_1890, and Mpro-3WL) and the Apo form are shown in Figure 3. The stability of the complexes is directly related to the RMSD values, with lower deviation indicating greater structural stability. The range that justifies complex stability is approximately ∼3 Å (Rafique et al., 2023). Our results demonstrate that the average RMSD values for all complexes fall within the range of 1.47–2.14 Å, indicating that all complexes remain stable during the 100 ns simulation trajectory (Figure 3).

The average RMSD values for the Apo protein were 1.89 Å, 2.04 Å for the reference complex (Mpro-3WL), 1.47 Å for Mpro-NuBBE_867, and 2.14 Å for Mpro-NuBBE_1890. The RMSD values revealed that the natural products (NPs) NuBBE_867 enhanced the structural stability of the Mpro protein (Figure 3), inhibiting its active site when compared to the crystallographic inhibitor used as a reference. On the other hand, NuBBE_1890 showed an average RMSD value higher than the Apo protein and the reference inhibitor, indicating that the presence of the ligand influenced the stability of the Mpro enzyme and altered its dynamic behavior. The results of RMSD values showed that the inhibitors formed stable bonds with the active site residues of the protein.

**FIGURE 4:**
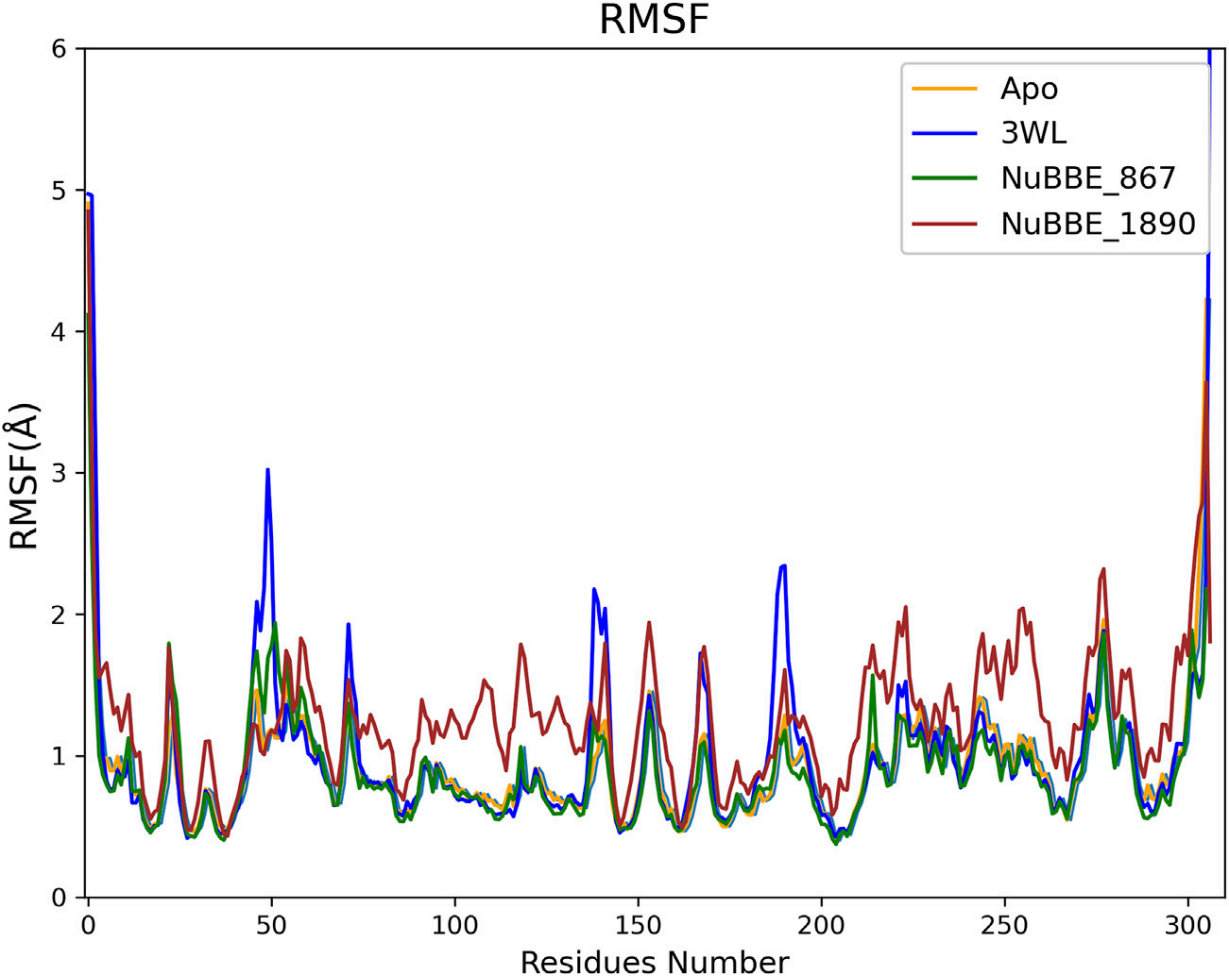
Root Mean Square Fluctuations of the Apo protein and Mpro-ligand complexes’ residues.

One essential component in determining the stability of a protein-ligand complex is the Root Mean Square Fluctuation (RMSF), which measures the flexibility of Cα atoms within a protein. Residues play a crucial role in forming a stable and strong binding complex between a protein and a ligand (Surti et al., 2020; Singh et al., 2022). The RMSF for the Apo protein and all simulated complexes are displayed in Figure 4.

The RMSF of 3WL showed slightly higher fluctuations when compared to the other simulated systems, suggesting that this system is less stable than the others, a hypothesis that is later supported by the free energy calculations. In general, regions with lower fluctuations included the active site regions of Mpro, including Glu166, Cys145, Asn142, Phe140, Met165, Met49, His41, and Leu141, which comprise more stable regions. In regions where the simulated ligand interacts with amino acid residues of the protein, lower fluctuation values indicate greater stability in those interactions. Particularly noteworthy are the regions of the Ser46, Met49, Cys145, and Gln189 residues.

The fluctuations of the binding site residues Glu166, Cys145, Asn142, Phe140, Met165, Met49, His41, and Leu141 were lower in all Mpro complexes. Small fluctuations in these binding site residues indicated high stability of the Mpro complexes. Furthermore, the data suggested that the residues involved in the binding were responsible for the stable RMSD of the Mpro complexes.

### Hydrogen bonding

The analysis of hydrogen bonds is an essential parameter that provides information about the binding affinity of drug candidates to a protein. Therefore, the presence of a significant number of H-bonds indicates a strong interaction between a ligand-protein complex. It is also responsible for drug metabolism and specificity (Rafique et al., 2023).

**FIGURE 5:**
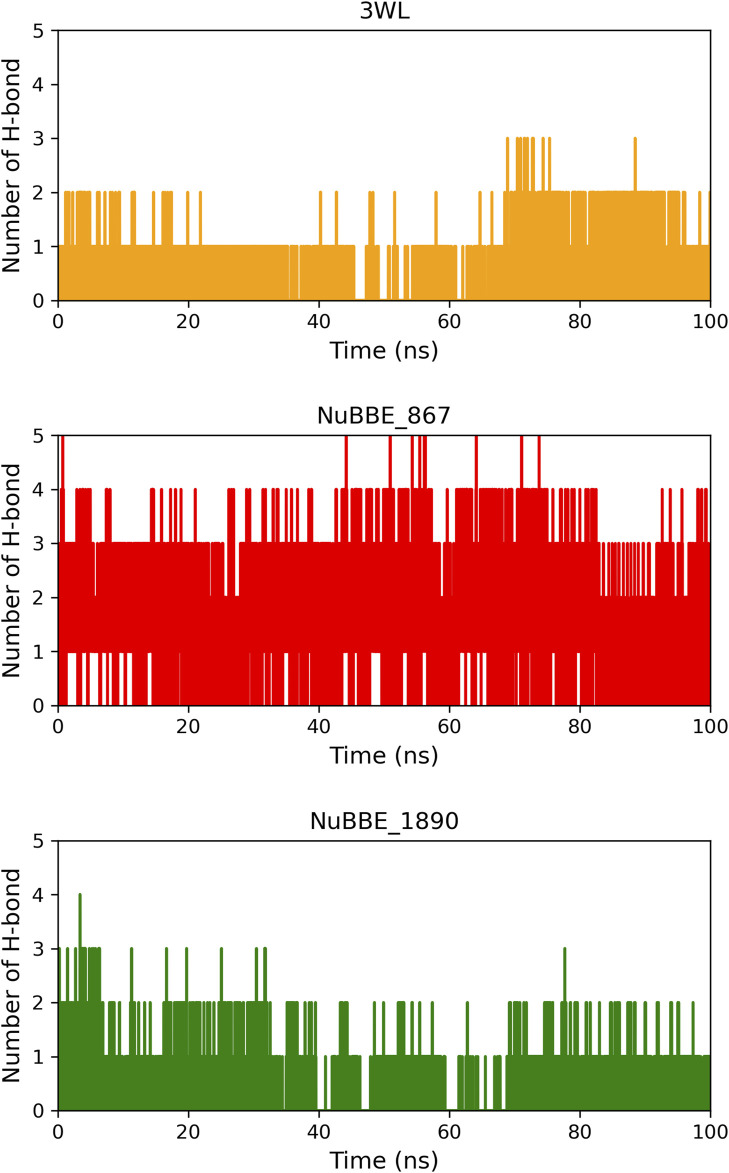
Number of hydrogen bonds between the ligands and Mpro protein during the 100 ns MD simulation time.

We investigated the hydrogen interactions of the flavonoids NuBBE_867 and NuBBE_1890 with the Mpro protein’s site residues and compared them to the crystallographic inhibitor 3WL during a 100 ns MD simulation (Figure 5).

Our results show that NuBBE_867 formed five hydrogen bonds with the active site of Mpro throughout the simulation, a higher number of interactions compared to NuBBE_1890 and the crystallographic inhibitor 3WL, which had three and two interactions throughout the simulation, respectively.

During the simulation period, NuBBE_867 interacted with the site residues Asp187, Glu166, Ser144, Gly143, and Cys145 with occupancies of 54.19%, 43.06%, 12.28%, 12.24%, and 11.48%, respectively. NuBBE_1890 interacted with the residues Asn142, Thr24, and Glu166 with occupancies of 17.82%, 7.89%, and 6.29%, respectively. In contrast, the crystallographic inhibitor interacted with the residues Arg188 and Glu166 with occupancies of 21.17% and 11.10%, respectively. The formation of multiple hydrogen bonds (H-bonds) between the NuBBE_867 inhibitor and the Mpro protein contributed to the greater stabilization of the structural complex, as shown in the RMSD analysis. The residues Gly143, Ser144, and Cys145 that interacted with NuBBE_867 constitute the “oxyanion hole” of this cysteine protease and play a fundamental role in catalyzing the hydrolysis of protein substrates, in line with studies on Mpro inhibitors by flavonoids (Lin et al., 2023). It is important to note that the interactions with the residues Cys145 and Glu166 are consistent with molecular docking. The Cys25 residue is essential for the catalytic function of Mpro, while the Glu166 residue is crucial for Mpro dimerization and the formation of the substrate binding pocket (Cherrak et al., 2020).

In a previous study, Yoshino and colleagues ((Yoshino et al., 2020) conducted molecular dynamics simulations involving different inhibitors complexed with Mpro to verify the necessary interactions with the active site residues of Mpro for considering a compound as an inhibitor. They revealed that residues Glu166, Gly143, Ser144, and Cys145 were among the main interacting residues, which corroborates our results. Similar interactions have also been observed in previous studies involving natural flavonoids, further reinforcing our findings (Cherrak et al., 2020; Su et al., 2020; Lin et al., 2023).

Our results demonstrate that NuBBE_867 effectively interacted with the binding pocket of the Mpro protein, is highly stable, and is expected to be a potential candidate as an inhibitor of the main protease of SARS-CoV-2.

### MM/PBSA binding free energy

To confirm the inhibitory capacity of the ligands for the Mpro protein in the ligand-protein complexes, binding free energy calculations were performed. The calculated results of MM-PBSA binding free energy are presented in Table 3.

**TABLE 3:** MM-PBSA binding free energies (∆*G*_bind_) and their components for the complexes under study. All values are reported in kcal.mol^−1^.

Parameters energy (kcal/mol)	Enzyme-ligand complexes		
	Mpro-3WL	Mpro-NuBBE_867	Mpro-NuBBE_1890
Van de Waals	−27.79	−41.18	−40.80
Electrostatic	−7.23	32.25	−18.21
Polar solvation	21.89	47.35	38.36
SASA	−2.84	−3.96	−4.26
Total Binding Free Energy	−15.97	−30.04	−24.92

The MM-PBSA calculations results for NuBBE_867, NuBBE_1890, and the inhibitor 3WL are shown in Table 3. From the results, the ∆Gbind of the Mpro-3WL complex was −15.97 kcal/mol. The ∆Gbind of Mpro-NuBBE_867 and Mpro-NuBBE_1890 was −30.04 kcal/mol and −24.92 kcal/mol, respectively. It was observed from the MM-PBSA analysis that the complexes formed between the flavonoids and Mpro had a lower ∆Gbind than the reference complex, Mpro-3WL. This indicates the formation of stable complexes with higher binding affinity for these flavonoids in the active site of Mpro. It's worth noting that NuBBE_867 exhibited the highest binding affinity to Mpro, suggesting a more potent inhibitor of Mpro when compared to the 3WL inhibitor.

Notably, these results are in line with the findings in the analysis of hydrogen bonds, which show that NuBBE_867 formed interactions with key residues in the active site of Mpro, explaining the higher affinity with Mpro. The MM-PBSA method has been effectively used in previous computational studies to estimate and select more potent inhibitors of the Mpro protein from natural sources, further supporting our results (Ahmad et al., 2021; Gogoi et al., 2021; Sharma et al., 2022; Lin et al., 2023; Rafique et al., 2023).

Additionally, energy terms such as van der Waals energy, electrostatic energy, SASA energy, and polar solvation energy are crucial contributors to the total binding free energy of the complexes. We can see in Table 3 that van der Waals, electrostatic, and SASA energies contributed significantly to the total binding energies of the complexes. On the other hand, polar solvation energy had an unfavorable impact on the binding energy.

**FIGURE 6:**
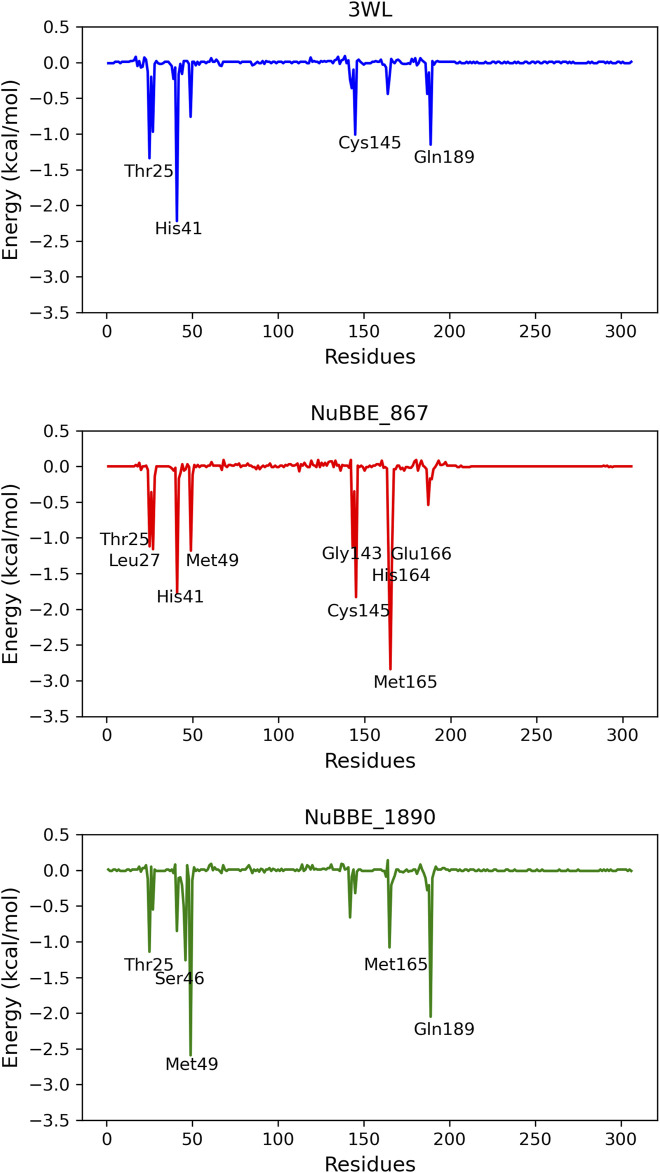
Graphical representation of the interaction energy per residue.

To delineate the specific contribution of each amino acid residue to ΔGbind, we conducted an energy decomposition analysis for each residue within the protein. Residues contributing to binding with values of −1.00 kcal/mol or lower were deemed significant for the binding process. The energetic contributions of each residue in the Mpro-NuBBE_867 and Mpro-NuBBE_1890 complexes were compared to those in the complexes formed by the reference compounds Mpro-3WL, as illustrated in Figure 6.

The energy decomposition analysis by residues unveiled that NuBBE_867 engages with His41 (ΔGtotal = −1.77) and Cys145 (ΔGtotal = −1.83), both of which constitute the catalytic dyad within this cysteine protease. Additionally, it forms interactions with Gly143 (ΔGtotal = −1.83), a residue that, alongside Cys145, plays a pivotal role in the oxyanion hole responsible for ligand stabilization. Moreover, it interacts with Glu166 (ΔGtotal = −1.14), which contributes to substrate recognition (Antonopoulou et al., 2022; Lin et al., 2023). Furthermore, it establishes connections with residues Thr25 (ΔGtotal = −1.12), Leu27 (ΔGtotal = −1.16), Met49 (ΔGtotal = −1.18), His164 (ΔGtotal = −1.43), and Met165 (ΔGtotal = −2.8). All these residues belong to the catalytic site and are classified as key residues of Mpro (Rampogu et al., 2023).

The residues contributing to the stability of NuBBE_1890 at the Mpro site are Thr25 (ΔGtotal = −1.10), Ser46 (ΔGtotal = −1.26), Met49 (ΔGtotal = −2.59), Met165 (ΔGtotal = −1.07), and Gln189 (ΔGtotal = −2.05), all situated in the active site of Mpro (Rampogu et al., 2023). In the case of the reference compound 3WL, the principal contributions to ΔGbind originated from residues Thr25 (ΔGtotal = −1.34), His41 (ΔGtotal = −2.21), Cys145 (ΔGtotal = −1.10), and Gln189 (ΔGtotal = −1.15).

Figure 6 depicts a greater number of residues with substantial contributions to the flavonoid NuBBE_867 in comparison to the reference ligands 3WL and NuBBE_1890. This observation elucidates the heightened binding affinity of this ligand with the Mpro enzyme, as indicated in Table 3, thus solidifying the validity of our findings.

Our *in silico* results propose that the molecule NuBBE_867 holds significant promise as an inhibitor of the Mpro enzyme.

## Conclusion

We have shown that Flavonoids may be effective to inhibit the SARS-CoV-2 Mpro protein. Molecular docking results indicate that all the compounds exhibit reasonably strong binding energies, ranging from −5.9 to −7.3 kcal/mol, and lower predicted inhibition constants compared to two FDA-approved antiviral drugs, remdesivir and nirmatrelvir, currently used in SARS-CoV-2 treatment. Subsequent MD simulations confirm the stability of the ligand-protein complexes. Thus, RMSD, RMSF, radius of gyration, and hydrogen bond interactions demonstrate the maintenance of structural stability and productive interactions. In conclusion, based on the collective findings of this study, it is evident that these compounds hold promise as candidates for the development of effective medications to inhibit the SARS-CoV-2 Mpro protein. Further *in vitro* and *in vivo* experiments are warranted to validate and advance these results, potentially contributing to the development of novel antiviral agents.

## Data Availability

The datasets presented in this study can be found in online repositories. The names of the repository/repositories and accession number(s) can be found in the article/Supplementary Material.

## References

[B1] Aanouz I. Belhassan A. El-Khatabi K. Lakhlifi T. El-Ldrissi M. Bouachrine M. (2021). Moroccan Medicinal plants as inhibitors against SARS-CoV-2 main protease: computational investigations. J. Biomol. Struct. Dyn. 39, 2971–2979. 10.1080/07391102.2020.1758790 32306860 PMC7212546

[B2] Ahamed A. Arif I. A. (2023). Finding potential inhibitors for Main protease (Mpro) of SARS-CoV-2 through Virtual screening and MD simulation studies. Saudi J. Biol. Sci. 30, 103845. 10.1016/j.sjbs.2023.103845 38020225 PMC10663854

[B3] Ahmad S. Abbasi H. W. Shahid S. Gul S. Abbasi S. W. (2021). Molecular docking, simulation and MM-PBSA studies of nigella sativa compounds: a computational quest to identify potential natural antiviral for COVID-19 treatment. J. Biomol. Struct. Dyn. 39, 4225–4233. 10.1080/07391102.2020.1775129 32462996 PMC7298883

[B4] Alzaabi M. M. Hamdy R. Ashmawy N. S. Hamoda A. M. Alkhayat F. Khademi N. N. (2022). Flavonoids are promising safe therapy against COVID-19. Phytochem. Rev. 21, 291–312. 10.1007/s11101-021-09759-z 34054380 PMC8139868

[B5] Antonio A. da S. Wiedemann L. S. M. Veiga-Junior V. F. (2020). Natural products’ role against COVID-19. RSC Adv. 10, 23379–23393. 10.1039/D0RA03774E 35693131 PMC9122563

[B6] Antonopoulou I. Sapountzaki E. Rova U. Christakopoulos P. (2022). Inhibition of the main protease of SARS-CoV-2 (Mpro) by repurposing/designing drug-like substances and utilizing nature’s toolbox of bioactive compounds. Comput. Struct. Biotechnol. J. 20, 1306–1344. 10.1016/j.csbj.2022.03.009 35308802 PMC8920478

[B7] Asselah T. Durantel D. Pasmant E. Lau G. Schinazi R. F. (2021). COVID-19: discovery, diagnostics and drug development. J. Hepatology 74, 168–184. 10.1016/j.jhep.2020.09.031 PMC7543767 33038433

[B8] Braz Filho R. Gottlieb O. R. (1971). The flavones of Apuleia leiocarpa. Phytochemistry 10, 2433–2450. 10.1016/S0031-9422(00)89891-X

[B9] Case D. A. Aktulga H. M. Belfon K. Cerutti D. S. Cisneros G. A. Cruzeiro V. W. D. (2023). AmberTools. J. Chem. Inf. Model 63, 6183–6191. 10.1021/acs.jcim.3c01153 37805934 PMC10598796

[B10] Cataneo A. H. D. Kuczera D. Koishi A. C. Zanluca C. Silveira G. F. Arruda T. B. de (2019). The citrus flavonoid naringenin impairs the *in vitro* infection of human cells by Zika virus. Sci. Rep. 9, 16348. 10.1038/s41598-019-52626-3 31705028 PMC6841724

[B11] Chatterjee S. Pyne N. Paul S. (2023). *In silico* screening of flavonoids unearthed Apigenin and Epigallocatechin Gallate, possessing antiviral potentiality against Delta and Omicron variants of SARS-CoV-2. Nucleus. 10.1007/s13237-023-00431-9

[B12] Chauhan S. (2020). Comprehensive review of coronavirus disease 2019 (COVID-19). Biomed. J. 43, 334–340. 10.1016/j.bj.2020.05.023 32788071 PMC7263230

[B13] Cherrak S. A. Merzouk H. Mokhtari-Soulimane N. (2020). Potential bioactive glycosylated flavonoids as SARS-CoV-2 main protease inhibitors: a molecular docking and simulation studies. PLoS One 15, e0240653. 10.1371/journal.pone.0240653 33057452 PMC7561147

[B14] Costa R. A. D. Costa A. D. S. S. D. Rocha J. A. P. D. Lima M. R. D. C. Rocha E. C. M. D. Nascimento F. C. A. (2023). Exploring natural alkaloids from Brazilian biodiversity as potential inhibitors of the *Aedes aegypti* juvenile hormone enzyme: a computational approach for vector mosquito control. Molecules 28, 6871. 10.3390/molecules28196871 37836714 PMC10574778

[B15] Costa R. A. da Rocha J. A. P. da Pinheiro A. S. Costa A. do S. S. D. Rocha E. C. M. da Josino L. P. C. (2022). *In silico* identification of novel allosteric inhibitors of Dengue virus NS2B/NS3 serine protease: scientific paper. J. Serbian Chem. Soc. 87, 693–706. 10.2298/JSC210929011D

[B16] Darden T. York D. M. Pedersen N. L. (1993). Particle mesh Ewald: an N⋅log(N) method for Ewald sums in large systems. J. Chem. Phys. 98, 10089–10092. 10.1063/1.464397

[B17] de Sousa L. R. F. Ramalho S. D. Burger M. C. de M. Nebo L. Fernandes J. B. da Silva M. F. (2014). Isolation of arginase inhibitors from the bioactivity-guided fractionation of byrsonima coccolobifolia leaves and stems. J. Nat. Prod. 77, 392–396. 10.1021/np400717m 24521209

[B18] dos Santos D. A. P. Braga P. A. de C. da Silva M. F. das G. F. Fernandes J. B. Vieira P. C. (2009). Anti-African trypanocidal and antimalarial activity of natural flavonoids, dibenzoylmethanes and synthetic analogues. J. Pharm. Pharmacol. 61, 257–266. 10.1211/jpp.61.02.0017 19178775

[B19] Duong C. Q. Nguyen P. T. V. (2023). Exploration of SARS-CoV-2 Mpro noncovalent natural inhibitors using structure-based approaches. ACS Omega 8, 6679–6688. 10.1021/acsomega.2c07259 36844600 PMC9947982

[B20] Ferracin R. J. das G. F. da Silva M. F. Fernandes J. B. Vieira P. C. (1998). Flavonoids from the fruits of Murraya paniculata. Phytochemistry 47, 393–396. 10.1016/S0031-9422(97)00598-0

[B21] Gahlawat A. Kumar N. Kumar R. Sandhu H. Singh I. P. Singh S. (2020). Structure-based virtual screening to discover potential lead molecules for the SARS-CoV-2 main protease. J. Chem. Inf. Model. 60, 5781–5793. 10.1021/acs.jcim.0c00546 32687345

[B22] Genheden S. Ryde U. (2015). The MM/PBSA and MM/GBSA methods to estimate ligand-binding affinities. Expert Opin. Drug Discov. 10, 449–461. 10.1517/17460441.2015.1032936 25835573 PMC4487606

[B23] Gogoi N. Chowdhury P. Goswami A. K. Das A. Chetia D. Gogoi B. (2021). Computational guided identification of a citrus flavonoid as potential inhibitor of SARS-CoV-2 main protease. Mol. Divers 25, 1745–1759. 10.1007/s11030-020-10150-x 33236176 PMC7685905

[B24] Gordon J. C. Myers J. B. Folta T. Shoja V. Heath L. S. Onufriev A. (2005). H++: a server for estimating pKas and adding missing hydrogens to macromolecules. Nucleic Acids Res. 33, W368–W371. 10.1093/nar/gki464 15980491 PMC1160225

[B25] Goyal B. Goyal D. (2020). Targeting the dimerization of the main protease of coronaviruses: a potential broad-spectrum therapeutic strategy. ACS Comb. Sci. 22, 297–305. 10.1021/acscombsci.0c00058 32402186

[B26] Heaton P. M. (2020). The covid-19 vaccine-development multiverse. N. Engl. J. Med. 383, 1986–1988. 10.1056/NEJMe2025111 32663910 PMC7377255

[B27] Hu Q. Xiong Y. Zhu G.-H. Zhang Y.-N. Zhang Y.-W. Huang P. (2022). The SARS-CoV-2 main protease (Mpro): structure, function, and emerging therapies for COVID-19. MedComm 3, e151. 10.1002/mco2.151 35845352 PMC9283855

[B28] Huang C. Shuai H. Qiao J. Hou Y. Zeng R. Xia A. (2023). A new generation Mpro inhibitor with potent activity against SARS-CoV-2 Omicron variants. Sig Transduct. Target Ther. 8, 128–213. 10.1038/s41392-023-01392-w PMC10018608 36928316

[B29] Islam M. T. Sarkar C. El-Kersh D. M. Jamaddar S. Uddin S. J. Shilpi J. A. (2020). Natural products and their derivatives against coronavirus: a review of the non-clinical and pre-clinical data. Phytotherapy Res. 34, 2471–2492. 10.1002/ptr.6700 32248575

[B30] Jackson L. A. Anderson E. J. Rouphael N. G. Roberts P. C. Makhene M. Coler R. N. (2020). An mRNA vaccine against SARS-CoV-2 - preliminary report. N. Engl. J. Med. 383, 1920–1931. 10.1056/NEJMoa2022483 32663912 PMC7377258

[B31] Jakalian A. Jack D. B. Bayly C. I. (2002). Fast, efficient generation of high-quality atomic charges. AM1-BCC model: II. Parameterization and validation. J. Comput. Chem. 23, 1623–1641. 10.1002/jcc.10128 12395429

[B32] Jin Z. Du X. Xu Y. Deng Y. Liu M. Zhao Y. (2020). Structure of Mpro from SARS-CoV-2 and discovery of its inhibitors. Nature 582, 289–293. 10.1038/s41586-020-2223-y 32272481

[B33] Jo S. Kim S. Shin D. H. Kim M.-S. (2020). Inhibition of SARS-CoV 3CL protease by flavonoids. J. Enzyme Inhibition Med. Chem. 35, 145–151. 10.1080/14756366.2019.1690480 PMC6882434 31724441

[B34] Kumar S. Sharma P. P. Shankar U. Kumar D. Joshi S. K. Pena L. (2020). Discovery of new hydroxyethylamine analogs against 3CLpro protein target of SARS-CoV-2: molecular docking, molecular dynamics simulation, and structure–activity relationship studies. J. Chem. Inf. Model. 60, 5754–5770. 10.1021/acs.jcim.0c00326 32551639

[B35] Li F. Wang Y. Li D. Chen Y. Dou Q. P. (2019). Are we seeing a resurgence in the use of natural products for new drug discovery? Expert Opin. Drug Discov. 14, 417–420. 10.1080/17460441.2019.1582639 30810395

[B36] Lin L. Chen D.-Y. Scartelli C. Xie H. Merrill-Skoloff G. Yang M. (2023). Plant flavonoid inhibition of SARS-CoV-2 main protease and viral replication. iScience 26, 107602. 10.1016/j.isci.2023.107602 37664626 PMC10470319

[B37] Lipinski C. A. Lombardo F. Dominy B. W. Feeney P. J. (2001). Experimental and computational approaches to estimate solubility and permeability in drug discovery and development settings 1PII of original article: S0169-409X(96)00423-1. The article was originally published in Advanced Drug Delivery Reviews 23 (1997) 3–25. 1. Adv. Drug Deliv. Rev. 46, 3–26. 10.1016/s0169-409x(00)00129-0 11259830

[B38] Liskova A. Samec M. Koklesova L. Samuel S. M. Zhai K. Al-Ishaq R. K. (2021). Flavonoids against the SARS-CoV-2 induced inflammatory storm. Biomed. Pharmacother. 138, 111430. 10.1016/j.biopha.2021.111430 33662680 PMC7906511

[B39] Liu H. Iketani S. Zask A. Khanizeman N. Bednarova E. Forouhar F. (2022). Development of optimized drug-like small molecule inhibitors of the SARS-CoV-2 3CL protease for treatment of COVID-19. Nat. Commun. 13, 1891. 10.1038/s41467-022-29413-2 35393402 PMC8989888

[B40] Maier J. A. Martinez C. Kasavajhala K. Wickstrom L. Hauser K. E. Simmerling C. (2015). ff14SB: improving the accuracy of protein side chain and backbone parameters from ff99SB. J. Chem. Theory Comput. 11, 3696–3713. 10.1021/acs.jctc.5b00255 26574453 PMC4821407

[B41] Mani J. S. Johnson J. B. Steel J. C. Broszczak D. A. Neilsen P. M. Walsh K. B. (2020). Natural product-derived phytochemicals as potential agents against coronaviruses: a review. Virus Res. 284, 197989. 10.1016/j.virusres.2020.197989 32360300 PMC7190535

[B42] Mohapatra P. K. Chopdar K. S. Dash G. C. Mohanty A. K. Raval M. K. (2023). *In silico* screening and covalent binding of phytochemicals of Ocimum sanctum against SARS-CoV-2 (COVID 19) main protease. J. Biomol. Struct. Dyn. 41, 435–444. 10.1080/07391102.2021.2007170 34821198

[B43] Mukae H. Yotsuyanagi H. Ohmagari N. Doi Y. Imamura T. Sonoyama T. (2022). A randomized phase 2/3 study of ensitrelvir, a novel oral SARS-CoV-2 3C-like protease inhibitor, in Japanese patients with mild-to-moderate COVID-19 or asymptomatic SARS-CoV-2 infection: results of the phase 2a part. Antimicrob. Agents Chemother. 66, e0069722. 10.1128/aac.00697-22 36098519 PMC9578433

[B44] Mukae H. Yotsuyanagi H. Ohmagari N. Doi Y. Sakaguchi H. Sonoyama T. (2023). Efficacy and safety of ensitrelvir in patients with mild-to-moderate coronavirus disease 2019: the phase 2b part of a randomized, placebo-controlled, phase 2/3 study. Clin. Infect. Dis. 76, 1403–1411. 10.1093/cid/ciac933 36477182 PMC10110269

[B45] Ngo S. T. Tam N. M. Pham M. Q. Nguyen T. H. (2021). Benchmark of popular free energy approaches revealing the inhibitors binding to SARS-CoV-2 Mpro. J. Chem. Inf. Model. 61, 2302–2312. 10.1021/acs.jcim.1c00159 33829781

[B46] Owen D. R. Allerton C. M. N. Anderson A. S. Aschenbrenner L. Avery M. Berritt S. (2021). An oral SARS-CoV-2 Mpro inhibitor clinical candidate for the treatment of COVID-19. Science 374, 1586–1593. 10.1126/science.abl4784 34726479

[B47] Owis A. I. El-Hawary M. S. Amir D. E. Refaat H. Alaaeldin E. Aly O. M. (2021). Flavonoids of Salvadora persica L. (meswak) and its liposomal formulation as a potential inhibitor of SARS-CoV-2. RSC Adv. 11, 13537–13544. 10.1039/D1RA00142F 35423847 PMC8697627

[B48] Panda S. K. Gupta P. S. S. Rana M. K. (2023). Potential targets of severe acute respiratory syndrome coronavirus 2 of clinical drug fluvoxamine: docking and molecular dynamics studies to elucidate viral action. Cell Biochem. Funct. 41, 98–111. 10.1002/cbf.3766 36478589

[B49] Patel C. N. Jani S. P. Jaiswal D. G. Kumar S. P. Mangukia N. Parmar R. M. (2021). Identification of antiviral phytochemicals as a potential SARS-CoV-2 main protease (Mpro) inhibitor using docking and molecular dynamics simulations. Sci. Rep. 11, 20295. 10.1038/s41598-021-99165-4 34645849 PMC8514552

[B50] Pilon A. C. Valli M. Dametto A. C. Pinto M. E. F. Freire R. T. Castro-Gamboa I. (2017). NuBBEDB: an updated database to uncover chemical and biological information from Brazilian biodiversity. Sci. Rep. 7, 7215. 10.1038/s41598-017-07451-x 28775335 PMC5543130

[B51] Poli G. Granchi C. Rizzolio F. Tuccinardi T. (2020). Application of MM-PBSA methods in virtual screening. Molecules 25, 1971. 10.3390/molecules25081971 32340232 PMC7221544

[B52] Price D. J. Brooks C. L. III (2004). A modified TIP3P water potential for simulation with Ewald summation. J. Chem. Phys. 121, 10096–10103. 10.1063/1.1808117 15549884

[B53] Purohit P. Sahoo P. S. Panda M. Kabasi K. Senapati S. K. Meher B. R. (2023). Evaluating the antiviral potential of phytocompounds from mesua ferrea against SARS-CoV-2 main protease: structure-based virtual screening and molecular dynamics simulation investigations. ChemistrySelect 8, e202302295. 10.1002/slct.202302295

[B54] Qiao J. Li Y.-S. Zeng R. Liu F.-L. Luo R.-H. Huang C. (2021). SARS-CoV-2 Mpro inhibitors with antiviral activity in a transgenic mouse model. Science 371, 1374–1378. 10.1126/science.abf1611 33602867 PMC8099175

[B55] Rafique A. Muhammad S. Iqbal J. Al-Sehemi A. G. Alshahrani M. Y. Ayub K. (2023). Exploring the inhibitory potential of novel piperidine-derivatives against main protease (Mpro) of SARS-CoV-2: a hybrid approach consisting of molecular docking, MD simulations and MMPBSA analysis. J. Mol. Liq. 382, 121904. 10.1016/j.molliq.2023.121904 37151376 PMC10131809

[B56] Rampogu S. Jung T. S. Ha M. W. Lee K. W. (2023). Repurposing and computational design of PARP inhibitors as SARS-CoV-2 inhibitors. Sci. Rep. 13, 10583. 10.1038/s41598-023-36342-7 37386052 PMC10310815

[B57] Rastelli G. Pinzi L. (2019). Refinement and rescoring of virtual screening results. Front. Chem. 7, 498. 10.3389/fchem.2019.00498 31355188 PMC6637856

[B58] Ray A. K. Sen Gupta P. S. Panda S. K. Biswal S. Bhattacharya U. Rana M. K. (2022). Repurposing of FDA-approved drugs as potential inhibitors of the SARS-CoV-2 main protease: molecular insights into improved therapeutic discovery. Comput. Biol. Med. 142, 105183. 10.1016/j.compbiomed.2021.105183 34986429 PMC8714248

[B59] Rubio-Martínez J. Jiménez-Alesanco A. Ceballos-Laita L. Ortega-Alarcón D. Vega S. Calvo C. (2021). Discovery of diverse natural products as inhibitors of SARS-CoV-2 Mpro protease through virtual screening. J. Chem. Inf. Model. 61, 6094–6106. 10.1021/acs.jcim.1c00951 34806382 PMC9931176

[B60] Ryckaert J.-P. Ciccotti G. Berendsen H. J. C. (1977). Numerical integration of the cartesian equations of motion of a system with constraints: molecular dynamics of n-alkanes. J. Comput. Phys. 23, 327–341. 10.1016/0021-9991(77)90098-5

[B61] Sahakyan H. (2021). Improving virtual screening results with MM/GBSA and MM/PBSA rescoring. J. Comput. Aided Mol. Des. 35, 731–736. 10.1007/s10822-021-00389-3 33983518

[B62] Salarizadeh N. Aallaei M. R. Zarei A. Malekshah R. E. Molaakbari E. Farajnezhadi A. (2022). Docking and molecular dynamics simulations of flavonoids as inhibitors of infectious agents: rutin as a coronavirus protease inhibitor. ChemistrySelect 7, e202202043. 10.1002/slct.202202043

[B63] Salomon-Ferrer R. Case D. A. Walker R. C. (2013). An overview of the Amber biomolecular simulation package. WIREs Comput. Mol. Sci. 3, 198–210. 10.1002/wcms.1121

[B64] Sander T. Freyss J. von Korff M. Rufener C. (2015). DataWarrior: an open-source program for Chemistry aware data visualization and analysis. J. Chem. Inf. Model. 55, 460–473. 10.1021/ci500588j 25558886

[B65] Sharma P. Joshi T. Mathpal S. Joshi T. Pundir H. Chandra S. (2022). Identification of natural inhibitors against Mpro of SARS-CoV-2 by molecular docking, molecular dynamics simulation, and MM/PBSA methods. J. Biomol. Struct. Dyn. 40, 2757–2768. 10.1080/07391102.2020.1842806 33143552 PMC7651194

[B66] Silvestrini L. Belhaj N. Comez L. Gerelli Y. Lauria A. Libera V. (2021). The dimer-monomer equilibrium of SARS-CoV-2 main protease is affected by small molecule inhibitors. Sci. Rep. 11, 9283. 10.1038/s41598-021-88630-9 33927258 PMC8085067

[B67] Singh R. Bhardwaj V. K. Das P. Bhattacherjee D. Zyryanov G. V. Purohit R. (2022). Benchmarking the ability of novel compounds to inhibit SARS-CoV-2 main protease using steered molecular dynamics simulations. Comput. Biol. Med. 146, 105572. 10.1016/j.compbiomed.2022.105572 35551011 PMC9052739

[B68] Solnier J. Fladerer J.-P. (2021). Flavonoids: a complementary approach to conventional therapy of COVID-19? Phytochem. Rev. 20, 773–795. 10.1007/s11101-020-09720-6 32982616 PMC7500502

[B69] Su H. Yao S. Zhao W. Li M. Liu J. Shang W. (2020). Anti-SARS-CoV-2 activities *in vitro* of Shuanghuanglian preparations and bioactive ingredients. Acta Pharmacol. Sin. 41, 1167–1177. 10.1038/s41401-020-0483-6 32737471 PMC7393338

[B70] Surti M. Patel M. Adnan M. Moin A. Ashraf S. A. Siddiqui A. J. (2020). Ilimaquinone (marine sponge metabolite) as a novel inhibitor of SARS-CoV-2 key target proteins in comparison with suggested COVID-19 drugs: designing, docking and molecular dynamics simulation study. RSC Adv. 10, 37707–37720. 10.1039/D0RA06379G 35515150 PMC9057143

[B71] Valli M. dos Santos R. N. Figueira L. D. Nakajima C. H. Castro-Gamboa I. Andricopulo A. D. (2013). Development of a natural products database from the biodiversity of Brazil. J. Nat. Prod. 76, 439–444. 10.1021/np3006875 23330984

[B72] Verdonk M. L. Cole J. C. Hartshorn M. J. Murray C. W. Taylor R. D. (2003). Improved protein-ligand docking using GOLD. Proteins 52, 609–623. 10.1002/prot.10465 12910460

[B73] Wang J. Wolf R. M. Caldwell J. W. Kollman P. A. Case D. A. (2004). Development and testing of a general amber force field. J. Comput. Chem. 25, 1157–1174. 10.1002/jcc.20035 15116359

[B74] Wang Z. Sun H. Yao X. Li D. Xu L. Li Y. (2016). Comprehensive evaluation of ten docking programs on a diverse set of protein–ligand complexes: the prediction accuracy of sampling power and scoring power. Phys. Chem. Chem. Phys. 18, 12964–12975. 10.1039/C6CP01555G 27108770

[B75] Wang Z. Yang L. (2020). Turning the tide: natural products and natural-product-inspired chemicals as potential counters to SARS-CoV-2 infection. Front. Pharmacol. 11, 1013. 10.3389/fphar.2020.01013 32714193 PMC7343773

[B76] Wu C. Yin W. Jiang Y. Xu H. E. (2022). Structure genomics of SARS-CoV-2 and its Omicron variant: drug design templates for COVID-19. Acta Pharmacol. Sin. 43, 3021–3033. 10.1038/s41401-021-00851-w 35058587 PMC8771608

[B77] Yoshino R. Yasuo N. Sekijima M. (2020). Identification of key interactions between SARS-CoV-2 main protease and inhibitor drug candidates. Sci. Rep. 10, 12493. 10.1038/s41598-020-69337-9 32719454 PMC7385649

[B78] Yu J. Tostanoski L. H. Peter L. Mercado N. B. McMahan K. Mahrokhian S. H. (2020). DNA vaccine protection against SARS-CoV-2 in rhesus macaques. Science 369, 806–811. 10.1126/science.abc6284 32434945 PMC7243363

[B79] Zakaryan H. Arabyan E. Oo A. Zandi K. (2017). Flavonoids: promising natural compounds against viral infections. Arch. Virol. 162, 2539–2551. 10.1007/s00705-017-3417-y 28547385 PMC7087220

[B80] Zhang L. Lin D. Sun X. Curth U. Drosten C. Sauerhering L. (2020). Crystal structure of SARS-CoV-2 main protease provides a basis for design of improved α-ketoamide inhibitors. Science 368, 409–412. 10.1126/science.abb3405 32198291 PMC7164518

