# Supplementary material for: Harnessing Brazilian biodiversity database: identification of flavonoids as potential inhibitors of SARS-CoV-2 main protease using computational approaches and all-atom molecular dynamics simulation
Source: Front Chem. 2024 Feb 22;12:1336001. doi: 10.3389/fchem.2024.1336001 (PMC10917896; doi:10.3389/fchem.2024.1336001)
Supplement: Supplementary file 1 [file Table1.DOCX]

Supplementary Material

Tabela S1: ΔGbind values obtained by the MM/GBSA method for all flavonoids used in this study.

| Ligands | MMGBSA (kcal/mol) |
| --- | --- |
| NuBBE_867 | -51,5700 |
| NuBBE_1884 | -50,1202 |
| NuBBE_1310 | -49,5517 |
| NuBBE_1890 | -48,2796 |
| NuBBE_2328 | -47,0772 |
| NuBBE_1880 | -45,9798 |
| NuBBE_1870 | -44,8566 |
| NuBBE_1878 | -44,1312 |
| NuBBE_1879 | -43,7034 |
| NuBBE_2007 | -42,6811 |
| NuBBE_1333 | -42,5288 |
| NuBBE_126 | -42,2776 |
| NuBBE_1694 | -41,9867 |
| NuBBE_1889 | -41,6262 |
| NuBBE_128 | -41,6000 |
| NuBBE_2172 | -41,3066 |
| NuBBE_2003 | -40,9515 |
| NuBBE_2417 | -40,7404 |
| NuBBE_1444 | -40,7235 |
| NuBBE_1888 | -40,6556 |
| NuBBE_1219 | -40,5976 |
| NuBBE_2001 | -40,0045 |
| NuBBE_1308 | -39,9722 |
| NuBBE_1864 | -39,8384 |
| NuBBE_2174 | -39,6073 |
| NuBBE_1871 | -39,6061 |
| NuBBE_1886 | -39,2091 |
| NuBBE_287 | -39,1142 |
| NuBBE_1222 | -39,1089 |
| NuBBE_1205 | -38,9831 |
| NuBBE_1883 | -38,8293 |
| NuBBE_1872 | -38,7946 |
| NuBBE_1215 | -38,7667 |
| NuBBE_2523 | -38,6801 |
| NuBBE_1226 | -38,2976 |
| NuBBE_2020 | -38,2747 |
| NuBBE_1189 | -38,2024 |
| NuBBE_1472 | -38,0646 |
| NuBBE_1814 | -37,9741 |
| NuBBE_1588 | -37,8712 |
| NuBBE_2522 | -37,7396 |
| NuBBE_2021 | -37,5849 |
| NuBBE_1224 | -37,5840 |
| NuBBE_2018 | -37,4563 |
| NuBBE_2004 | -37,4385 |
| NuBBE_1445 | -37,3497 |
| NuBBE_1867 | -37,2255 |
| NuBBE_1312 | -37,0784 |
| NuBBE_1306 | -36,8273 |
| NuBBE_1263 | -36,8071 |
| NuBBE_1589 | -36,7372 |
| NuBBE_2023 | -36,6255 |
| NuBBE_1311 | -36,6206 |
| NuBBE_1328 | -36,6186 |
| NuBBE_1316 | -36,5996 |
| NuBBE_1223 | -36,5458 |
| NuBBE_1693 | -36,5311 |
| NuBBE_1663 | -36,4961 |
| NuBBE_1873 | -36,4915 |
| NuBBE_1327 | -36,4608 |
| NuBBE_1875 | -36,4367 |
| NuBBE_1656 | -36,3525 |
| NuBBE_1225 | -36,3444 |
| NuBBE_2485 | -36,3140 |
| NuBBE_2427 | -36,2966 |
| NuBBE_2035 | -36,2510 |
| NuBBE_1305 | -36,1845 |
| NuBBE_2033 | -36,1613 |
| NuBBE_1559 | -36,1094 |
| NuBBE_1869 | -36,0911 |
| NuBBE_2000 | -36,0216 |
| NuBBE_1787 | -35,8936 |
| NuBBE_2083 | -35,8806 |
| NuBBE_1591 | -35,7800 |
| NuBBE_2081 | -35,7247 |
| NuBBE_1692 | -35,7010 |
| NuBBE_1625 | -35,6850 |
| NuBBE_178 | -35,6445 |
| NuBBE_1580 | -35,6129 |
| NuBBE_1221 | -35,5807 |
| NuBBE_2275 | -35,5734 |
| NuBBE_2434 | -35,5706 |
| NuBBE_1894 | -35,3891 |
| NuBBE_1893 | -35,0438 |
| NuBBE_2486 | -35,0431 |
| NuBBE_1586 | -34,7058 |
| NuBBE_2225 | -34,6383 |
| NuBBE_2175 | -34,5670 |
| NuBBE_2082 | -34,5665 |
| NuBBE_1318 | -34,5235 |
| NuBBE_2399 | -34,4895 |
| NuBBE_1868 | -34,4570 |
| NuBBE_122 | -34,4333 |
| NuBBE_1216 | -34,3931 |
| NuBBE_1874 | -34,3652 |
| NuBBE_1776 | -34,3132 |
| NuBBE_2049 | -34,2696 |
| NuBBE_1999 | -34,2229 |
| NuBBE_1443 | -34,1949 |
| NuBBE_1581 | -34,1847 |
| NuBBE_1322 | -34,1598 |
| NuBBE_2024 | -34,0285 |
| NuBBE_1592 | -34,0272 |
| NuBBE_2047 | -33,9968 |
| NuBBE_68 | -33,9066 |
| NuBBE_1865 | -33,9052 |
| NuBBE_1321 | -33,8114 |
| NuBBE_1590 | -33,7340 |
| NuBBE_2222 | -33,6109 |
| NuBBE_1895 | -33,6097 |
| NuBBE_2432 | -33,4515 |
| NuBBE_1777 | -33,2776 |
| NuBBE_320 | -33,2295 |
| NuBBE_2509 | -33,0321 |
| NuBBE_563 | -32,9910 |
| NuBBE_1073 | -32,8693 |
| NuBBE_2048 | -32,7572 |
| NuBBE_1558 | -32,6023 |
| NuBBE_1307 | -32,5845 |
| NuBBE_2526 | -32,5533 |
| NuBBE_1768 | -32,5281 |
| NuBBE_1309 | -32,5117 |
| NuBBE_2415 | -32,3545 |
| NuBBE_1220 | -32,3515 |
| NuBBE_1217 | -32,2932 |
| NuBBE_1218 | -32,2655 |
| NuBBE_2005 | -32,2605 |
| NuBBE_1695 | -31,9031 |
| NuBBE_1662 | -31,7896 |
| NuBBE_2524 | -31,7735 |
| NuBBE_293 | -31,7655 |
| NuBBE_2019 | -31,7438 |
| NuBBE_1997 | -31,7179 |
| NuBBE_1766 | -31,4219 |
| NuBBE_1557 | -31,3978 |
| NuBBE_2511 | -31,3426 |
| NuBBE_1765 | -31,2925 |
| NuBBE_1784 | -31,2613 |
| NuBBE_426 | -31,2344 |
| NuBBE_1815 | -31,2293 |
| NuBBE_2452 | -31,1620 |
| NuBBE_2431 | -31,0428 |
| NuBBE_180 | -30,9939 |
| NuBBE_139 | -30,9539 |
| NuBBE_2034 | -30,9315 |
| NuBBE_564 | -30,8886 |
| NuBBE_2022 | -30,8486 |
| NuBBE_67 | -30,6673 |
| NuBBE_1593 | -30,6275 |
| NuBBE_2450 | -30,4682 |
| NuBBE_1995 | -30,4555 |
| NuBBE_2508 | -30,3086 |
| NuBBE_1582 | -30,2449 |
| NuBBE_2006 | -30,0698 |
| NuBBE_1844 | -29,9986 |
| NuBBE_2416 | -29,9251 |
| NuBBE_2443 | -29,8957 |
| NuBBE_2413 | -29,7795 |
| NuBBE_1769 | -29,7714 |
| NuBBE_292 | -29,3012 |
| NuBBE_1320 | -29,2594 |
| NuBBE_562 | -29,1305 |
| NuBBE_1188 | -29,0932 |
| NuBBE_2224 | -29,0327 |
| NuBBE_2046 | -29,0252 |
| NuBBE_2080 | -28,9735 |
| NuBBE_2386 | -28,9697 |
| NuBBE_179 | -28,9201 |
| NuBBE_866 | -28,9115 |
| NuBBE_120 | -28,8815 |
| NuBBE_1996 | -28,7719 |
| NuBBE_1317 | -28,6687 |
| NuBBE_1908 | -28,6615 |
| NuBBE_355 | -28,6184 |
| NuBBE_1892 | -28,6044 |
| NuBBE_2173 | -28,0556 |
| NuBBE_2402 | -27,9687 |
| NuBBE_2025 | -27,9311 |
| NuBBE_1314 | -27,8261 |
| NuBBE_565 | -27,5631 |
| NuBBE_2430 | -27,5170 |
| NuBBE_2008 | -27,5055 |
| NuBBE_1560 | -27,5019 |
| NuBBE_1206 | -27,4505 |
| NuBBE_2036 | -27,3368 |
| NuBBE_2510 | -27,3228 |
| NuBBE_141 | -27,0677 |
| NuBBE_2282 | -27,0392 |
| NuBBE_2401 | -26,9075 |
| NuBBE_2279 | -26,8253 |
| NuBBE_1046 | -26,7558 |
| NuBBE_1562 | -26,2231 |
| NuBBE_1313 | -25,5619 |
| NuBBE_2280 | -25,0887 |
| NuBBE_177 | -24,8832 |
| NuBBE_2447 | -24,7817 |
| NuBBE_567 | -24,6827 |
| NuBBE_2448 | -24,0857 |
| NuBBE_2449 | -23,1450 |
| NuBBE_181 | -23,1124 |
| NuBBE_363 | -22,8868 |
| NuBBE_2451 | -20,4782 |
| NuBBE_1045 | -20,3413 |
| NuBBE_1866 | -11,8909 |
